# The Structural Basis of Localizing Polo-Like Kinase to the Flagellum Attachment Zone in *Trypanosoma brucei*

**DOI:** 10.1371/journal.pone.0027303

**Published:** 2011-11-11

**Authors:** Lu Sun, Ching C. Wang

**Affiliations:** Department of Pharmaceutical Chemistry, University of California San Francisco, San Francisco, California, United States of America; University of Georgia, United States of America

## Abstract

The polo-like kinase in the deep branching eukaryote *Trypanosoma brucei* (TbPlk) has many unique features. Unlike all the other polo-like kinases known to associate with the nucleus and controlling both mitosis and cytokinesis, TbPlk localizes to the flagellum attachment zone (FAZ) and regulates only cytokinesis in *T. brucei*. TbPlk was, however, previously found capable of complementing all the multiple Plk (Cdc5) functions in *Saccharomyces cerevisiae*, indicating that it has acquired all the functions of Cdc5. In the present study, Cdc5 tagged with an enhanced yellow fluorescence protein (EYFP) localized exclusively in the FAZ of *T. brucei*, suggesting that the unusual localization and limited function of TbPlk are probably attributed to the particular environment in *T. brucei* cells. Structural basis for the FAZ localization of TbPlk was further investigated with TbPlk and TbPlk mutants tagged with EYFP and expressed in *T. brucei*. The results indicated that a kinase-inactive mutant N169A and a TbPlk mutant with the entire kinase domain (KD) deleted both localized to the FAZ. Substantial association with FAZ was also maintained when one of the two polo-boxes (PB1 or 2) or the linker region between them was deleted from TbPlk. But a deletion of both polo-boxes led to a complete exclusion of the protein from FAZ. All the deletion mutants retained the kinase activity, further indicating that the TbPlk kinase function does not play a role for FAZ localization. The two polo boxes in TbPlk are most likely instrumental in localizing the protein to FAZ through potential interactions with certain FAZ structural component(s). A putative cryptic bipartite nuclear targeting signal was identified in TbPlk, which was capable of directing TbPlk into the nucleus when either the kinase activity was lost or the PB1 was deleted from the protein.

## Introduction

Polo-like kinases (Plks) are a family of conserved regulators of multiple events during cell division among the eukaryotes. They are associated with the nucleus most of the time throughout the cell cycle [1]. Their functions are required for centrosome duplication and maturation, DNA damage checkpoint activation, mitotic onset, bipolar spindle formation, Golgi fragmentation and assembly, chromosome segregation, and cytokinesis [1]–[4]. All the Plks share a highly conserved protein kinase domain in the amino-terminal region and a polo box domain (PBD) consisting of one or two structural motifs; the polo boxes (PB), in the noncatalytic carboxyl-terminal region [5]–[7].

Plk activity is regulated both in time and space throughout the cell cycle. Temporal control is achieved by transcriptional regulation, phosphorylation and proteolysis, Spatial control is achieved by interactions between Plks and other proteins that localize Plk to the appropriate site of action in due time [1]. It has been demonstrated that PBD allosterically inhibits the Plk activity by binding intra-molecularly to the kinase domain in the absence of bound substrate [8]. The inhibition is relieved by substrate binding to the active site, and the kinase activity is further enhanced by phosphorylation of the T210 residue in the T-loop of human Plk1 [9], [10]. Other than the function of self-inhibition, PBD is also thought to play a critical role in targeting Plks to specific subcellular structures to phosphorylate the substrates associated with the structure [7]. By analyzing various PBD mutants of Cdc5, the Plk in *Saccharomyces cerevisiae*, a mutation of three conserved amino acid residues (W517F/V518A/L530A) in polo box 1 (PB1) was sufficient to abolish the localization of Cdc5 at spindle poles and cytokinetic neck filaments without any change in its kinase activity [11], [12]. Additional evidence accumulated on the Plks from other organisms further confirmed that PBD is required for proper localizations and functions of Plk [11], [13]–[16]. A competition between an over-expressed PBD and Plk1 in mammalian cells interfered with the subcellular localization and mitotic functions of the latter [17], [18]. Thus, PBD dependent protein–protein interactions are apparently required for proper localization and function of Plks, though the proteins they interact with remain largely unidentified at the present time [19].

In a screening effort, a phospho-peptide with the sequence Pro-Met-Gln-Ser-pThr-Pro-Leu was found binding to the PBD of human Plk1 and disrupting Plk1 substrate binding and PBD-mediated localization to centrosomes [8]. The crystal structure of Plk1 PBD-phospho-peptide complex revealed that the two polo boxes exhibited similar folds with each comprising a six-stranded β sheet and an α-helix, forming a heterodimeric phospho-peptide-binding module, despite the low (∼12%) sequence identity between the two polo boxes [8], [20]. The phospho-peptide is located at the interface of the two polo boxes indicating contributions from both boxes for the binding. Four residues in PBD were found to dominate the interactions between PBD and the phospho-peptide: Trp414, Leu490, His538 and Lys540. They are conserved in the Plks of *S. cerevisiae*, *Drosophila melanogaster*, *Xenopus laevis*, *Caenorhabditis elegans* and mammals [21]. His538 and Lys540 in PB2 are essential for electrostatic interactions with the negatively charged phosphate group of the phosphorylated Thr (pThr) residue in the phospho-peptide, whereas Trp414 in PB1 is central for selecting the Ser residue at the pThr-1 position for hydrogen bonding and van der Waals interactions [22].

*Trypanosoma brucei* is an early-branching unicellular protozoan parasite that causes African sleeping sickness in humans and nagana in cattle. Its life cycle involves transmissions between the mammalian host and the tsetse flies. The parasites divide by binary fission from the anterior to the posterior end following the replications and segregations of nucleus, the single mitochondrion, the mitochondrial DNA complex known as the kinetoplast, the basal body, and the flagellum [23]. There is a flagellum attachment zone (FAZ) originating from the basal body and extending along the dorsal side of the cell to the anterior end of the cell [23]. During cell division, a new FAZ originates from the newly formed basal body in the posterior end and grows along the existing FAZ to directing the formation of a new cleavage furrow [24]. The chromosome passenger complex trans-localizes then from the midzone of nuclear central spindle to the FAZ during telophase and moves quickly to the anterior end of FAZ. This is followed by a rapid gliding of the complex along the FAZ toward the posterior end and a separation between the mother and daughter cells [25]. It represents a most unique mechanism of cytokinesis among the eukaryotes examined thus far.

There is a single Plk homologue in *T. brucei*, TbPlk [26]. Like the other Plks, it contains a conserved amino-terminal Ser/Thr kinase domain (KD), a potential bipartite nuclear localization signal (NLS) and a carboxy-terminal PBD with two polo boxes PB1 and 2 separated by a linker region. But it differs from all the other known Plks by localizing specifically to the FAZ and regulating only the cytokinesis in *T. brucei* [27]. It emerges in the basal body and the associated bilobed structure involved in Golgi duplication during S-phase [28]–[30]. It then migrates to the mid-point of FAZ during early mitosis and disperses into the cytoplasm during anaphase before the chromosome passenger complex is to trans-localize to the FAZ [31]. The dynamics of this trans-localization [30] is apparently unaffected by tagging the C-terminus of TbPlk with 3HA [27] or EYFP [28], thus facilitates further investigations. The association between TbPlk and FAZ is very strong and capable of withstanding detergent and high salt treatment [27]. TbPlk is the only Plk identified thus far that has a most unusual subcellular localization and a limited function in controlling only cytokinesis.

TbPlk lacks all the crucial amino acid residues in PBD required for localizing Cdc5 at the spindle poles and cytokinetic neck filaments in *S. cerevisiae* [11], [12] or interactions with the phospho-peptide in human Plk1 [21]. This could explain why TbPlk is not associated with the nucleus of *T. brucei*. Its exclusive localization to the FAZ may prevent it from exerting any regulatory function on mitosis. In an earlier study, we demonstrated that TbPlk was capable of complementing *S. cerevisiae* depleted of Cdc5, indicating that TbPlk possesses all the multiple activities of Cdc5 in controlling both mitosis and cytokinesis in yeast [27]. It suggested also that TbPlk was apparently distributed to the spindle poles and cytokinetic neck filaments in yeast like Cdc5 in order to exert the same multiple functions, in spite of its unusual localization to the FAZ in *T. brucei*. It could be the particular cellular environments existing in the two different types of cells that dictate the distinct locations of TbPlk.

Since the localization of over-expressed recombinant TbPlk [27] was found to be the same as that of endogenously expressed TbPlk in *T. brucei* [28], we over-expressed Cdc5 tagged with enhanced yellow fluorescence protein at its C-terminus (Cdc5-EYFP) in *T. brucei* and found that it was exclusively localized to the FAZ. We observed also that the kinase activity of TbPlk was not involved but the two polo boxes PB1 and PB2 were necessary for localizing the protein to FAZ. TbPlk is capable of entering the nucleus of *T. brucei*, but is apparently blocked by its own kinase activity and the presence of PB1 in the protein.

## Results

### Cdc5-EYFP expressed in *T. brucei* is localized to the FAZ

**Figure 1:**
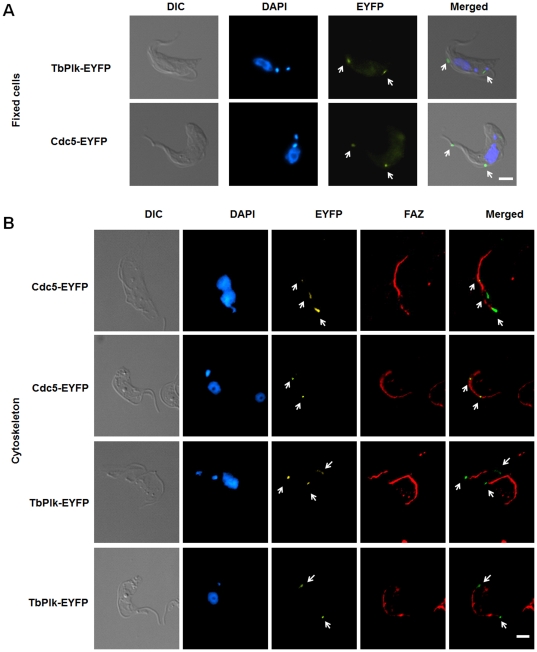
Both TbPlk-EYFP and Cdc5-EYFP localize to the FAZ in *T. brucei*. (A) Procyclic-form 29-13 cells harboring pLew100-TbPlk-EYFP or pLew100-Cdc5-EYFP were cultured for 1 day in the presence of 1.0 µg/ml tetracycline, fixed with 3.7% formaldehyde, and stained with DAPI. (B) The cytoskeletons were stained with L3B2 antibody for FAZ (red), and DAPI for DNA (blue). The results were from three independent experiments. Arrows indicate the TbPlk-EYFP and Cdc5-EYFP signals. Bars: 2 µm.

Cdc5 is localized to the spindle pole bodies at all times and at the mother budding neck region during mitosis and cytokinesis of *S. cerevisiae*. It is also seen as a diffused nuclear protein except in the G1 phase of yeast [1]. Cdc5 tagged with EYFP at the C-terminus (Cdc5-EYFP) was expressed in the insect form (procyclic form) of *T. brucei* strain 29–13 and its subcellular distribution was monitored with fluorescence microscopy (see Materials and Methods). The result indicated that many of the transfected cells contained two spots of fluorescence, one at the dorsal side and the other at the anterior tip of the cell (Figure 1A). This pattern is similar to that of that of TbPlk tagged at the C-terminus with either 3 hemmaglutinin epitopes (TbPlk-3HA) [27] or EYFP (TbPlk-EYFP) (Figure 1A, arrows), except that the latter was apparently caught during the dynamic trans-localization when both fluorescent spots were in the mid-portion of the dorsal side of the cell. Further treatment of the Cdc5-EYFP transfected cells with detergents and high salts isolated the FAZ, which was stained with the L3B2 antibody (a gift from Dr. Keith Gull of Oxford University) and identified in an immunofluorescence assay (Figure 1B). Two microscopic photographs are presented in the upper panel of Figure 1B, in which the micrograph above has two closely parallel FAZs with Cdc5-EYFP localized to the posterior end. The one below shows a single FAZ, where two Cdc5-EYFP spots are localized to its mid-portion. This pattern of localization of Cdc5-EYFP in FAZ is similar to that of TbPlk-EYFP in similar experiments (Figure 1B, lower panel, arrows), in which each of the two FAZs in the photograph on top has a TbPlk-EYFP at the posterior end, whereas only one of the two has also a cluster of TbPlk-EYFP at the anterior end. The other one either misses the anterior TbPlk-EYFP or has the spot out of focus in the photograph. The single FAZ in the lower picture has two fluorescent spots at the anterior and posterior ends respectively (Figure 1B, arrows). These photographs reflect the dynamic nature of Cdc5 and TbPlk localizations to the FAZ. The previously identified crucial residues in Cdc5 required for the vastly different subcellular distribution in *S. cerevisiae* [11] are probably not involved in the FAZ localization of Cdc5 in *T. brucei*, because they are not present in TbPlk.

### The kinase activity of TbPlk is not required for FAZ localization

**Figure 2:**
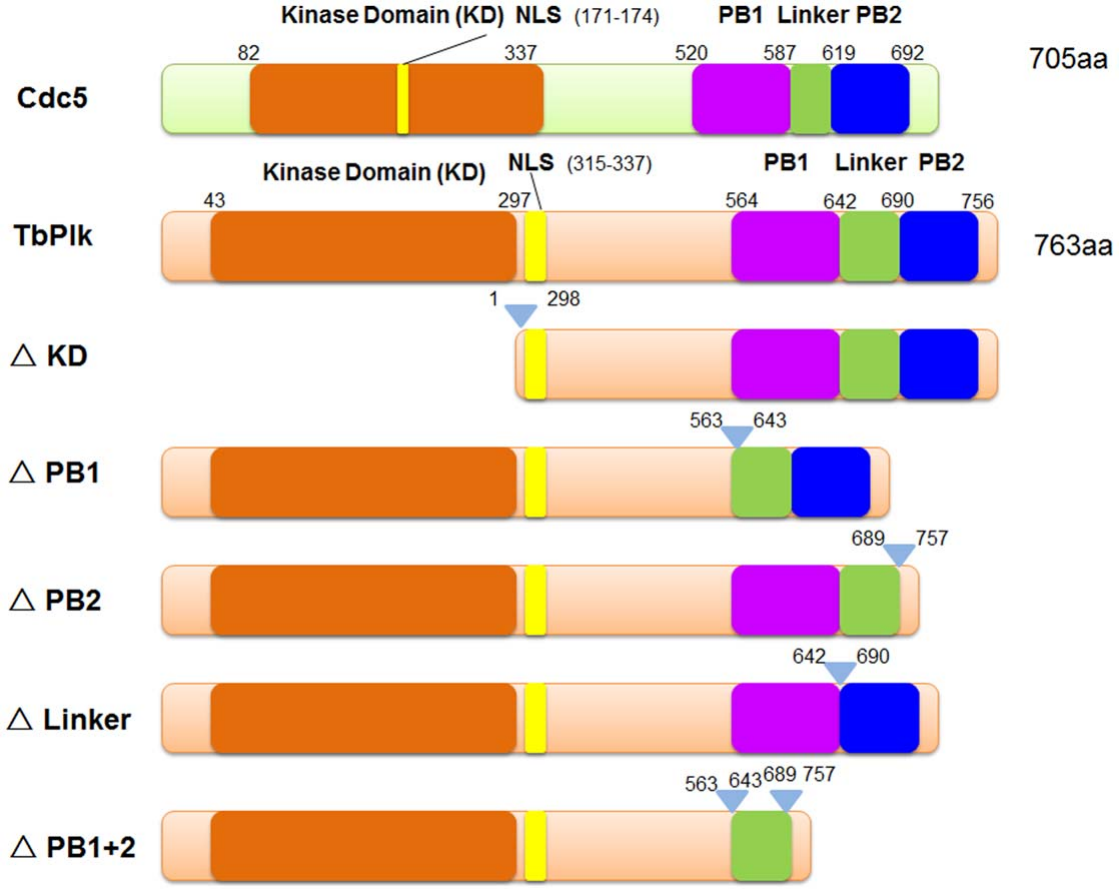
Schematic presentation of the primary structures of Cdc5, TbPlk and TbPlk mutants. NLS represents nuclear localization signal. PB represents polo box.

**Figure 3:**
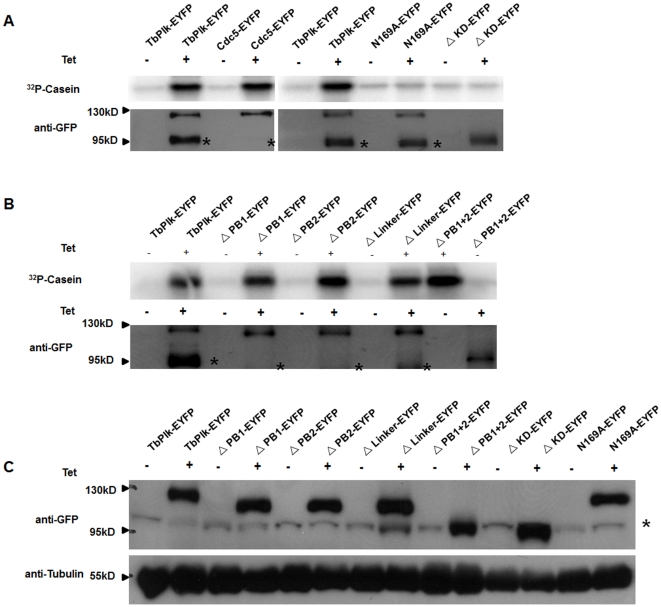
Assay of kinase activities in Cdc5-EYFP, TbPLK-EYFP and the mutants of TbPlk-EYFP. (A) The kinase activity of Cdc5-EYFP, TbPlk-EYFP, ΔKD-EYFP and N169A-EYFP. (B) The kinase activity of ΔPB1-EYFP, ΔPB2-EYFP, ΔLinker-EYFP, ΔPB1+2-EYFP mutants. All the fusion proteins were immunoprecipitated from the cell lysate with anti-GFP beads. The immunoprecipitates were quantified in Western blots and each incubated with 10 µg dephosphorylated casein and 5 μCi [γ-^32^P]ATP at 37°C for 1 hr. (C) Crude cell lysates of the transfected cell lines were analyzed on a Western blot stained with anti-GFP antibody. α-tubulin was stained with anti-tubulin antibody and used as a loading control. All the studies were derived from three independent experiments. The asterisks next to the Western blot indicate a nonspecific protein band.

TbPlk and Cdc5 have similar, albeit minutely distinctive primary structures (Figure 2). Each protein has a kinase domain (KD), a putative bipartite nuclear localizing signal (NLS) [32], PB1, the linker and PB2. Their detailed sequence alignment is presented in Figure S1. The localization of TbPlk-EYFP and Cdc5-EYFP to the FAZ did not reveal whether the kinase activities of the proteins are playing any pivotal role in the process of protein positioning (Figure 2). In the current study we performed kinase assays on TbPlk-EYFP and Cdc5-EYFP isolated from the lysates of *T. brucei* cells. The pLew100-TbPlk-EYFP or pLew100-Cdc5-EYFP transfected procyclic form *T. brucei* cells were cultured and induced with tetracycline (1 µg/ml) to express TbPlk-EYFP or Cdc5-EYFP. The fusion proteins were immunoprecipitated from the lysates with anti-GFP antibodies and were quantified in Western blot to similar levels. These samples were then assayed for their kinase activities using γ-^32^P-ATP and casein as substrates. The results showed that both TbPlk-EYFP and Cdc5-EYFP possess similarly substantial levels of kinase activities (Figure 3A). EYFP tagging at the C-termini of the kinase proteins thus does not interfere with their kinase functions.

**Figure 4:**
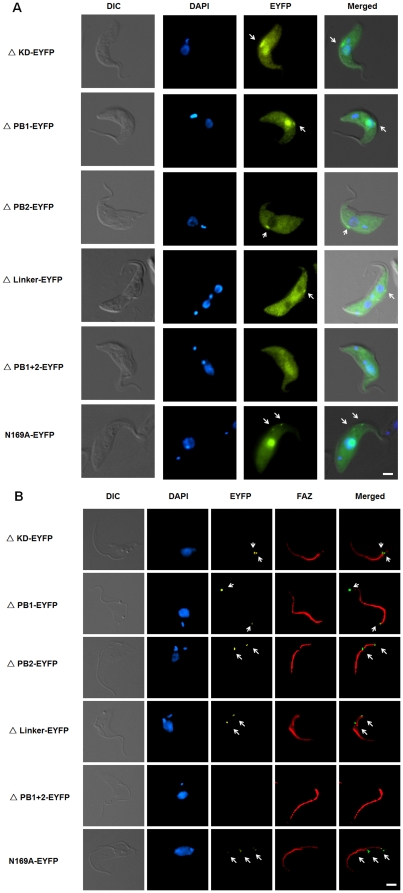
Localization of TbPlk-EYFP mutants in the transfected *T. brucei* cells and the cytoskeleton isolated from them. (A) Procyclic-form 29-13 cells expressing individual TbPlk-EYFP mutants were each cultured for 1 day in the presence of 1.0 µg/ml tetracycline, fixed with 3.7% formaldehyde, stained with DAPI and examined with fluorescence microscopy. (B) The cytoskeletons of the transfected cells were isolated after detergent and high salt treatment. They were stained with the L3B2 antibody to FAZ (red) and DAPI for DNA (blue) and observed with fluorescence microscopy. The results were from three independent experiments. Arrows indicate the signals of TbPlk-EYFP mutants. Bars: 2 µm.

N169A is a kinase-dead mutant of TbPlk [29]. We constructed the plasmid encoding N169A-EYFP and expressed the mutant protein in the transfected *T. brucei* as previously described. Enzyme assay showed that it has no detectable kinase activity (Figure 3A). But fluorescence microscopy indicated that the mutant protein localizes to the FAZ among the transfected cells (Figures 4A & 4B, arrows), though fluorescence was also detected in the cytoplasm and nucleus of the cells, suggesting compromised efficiency in FAZ targeting (Figure 4A and Table 1). A deletion mutant in which the entire kinase domain was deleted from TbPlk-EYFP (ΔKD-EYFP) (amino acid residues 1 to 298 were deleted, Figure 2) was also constructed and expressed in *T. brucei*. The truncated protein showed no kinase activity (Figure 3A). It localized, however, to the FAZ, cytoplasm and nucleus of all the transfected cells as well (Figures 4A & 4B; Table 1). The kinase activity of TbPlk is thus not essential for FAZ localization. But it can be apparently helpful in promoting more efficient trans-localization of TbPlk from cytoplasm to FAZ. Most interestingly, substantial N169A-EYFP and ΔKD-EYFP were found to localize to the nucleus among 73% and 100% of the transfected cells, respectively (Table 1). Thus, the kinase activity of TbPlk is apparently capable of inhibiting import of the protein into the nucleus, where all the other Plks in other eukaryotes are known to localize (see Discussion).

**Table 1:** Localization of TbPlk-EYFP and its mutants in *T. brucei*.

	FAZ	Nucleus	Cytoplasm
TbPlk	100%	0%	0%
Δ KD	100%	100%	100%
Δ PB1	100%	78%	100%
Δ PB2	83%	0%	100%
Δ Linker	56%	0%	100%
Δ PB1+2	0%	82%	100%
N169A	100%	73%	100%

Approximately 200 cells of each transfected cell line were examined with a fluorescence microscope. Percentages of cells with the fluorescent fusion protein localized to FAZ, nucleus and/or cytoplasm were tabulated from three independent experiments.

### Both polo boxes in TbPlk are required for localizing the protein to FAZ

In order to find out if any of the other structural motifs in TbPlk could play a role in localizing the protein to FAZ, we prepared a series of deletion mutant constructs of TbPlk tagged with EYFP at the C-terminus and expressed each of them in transfected *T. brucei* cells. They included the PB1-deletion mutant (ΔPB1-EYFP), the PB2-deletion mutant (ΔPB2-EYFP), the linker-deletion mutant (ΔLinker-EYFP) and the double PB1 and PB2 deletion mutant (ΔPB1+2-EYFP) (see Figure 2). The immunoprecipitated mutant proteins were quantified in Western blot to similar levels and assayed for kinase activity using casein as substrate, and all the mutants were found to possess similar activities equivalent to that of the wild-type TbPlk-EYFP (Figure 3B). These deleted domains are apparently not playing essential roles for the kinase function of TbPlk in the present assay.

In order to rule out the possibility that the mutant proteins may have different efficiencies of being immunoprecipitated with the anti-GFP antibody, thus reflecting unequal levels of expression in *T. brucei* cells, we monitored the levels of mutant proteins in the respective crude cell lysates with a Western. The results in Figure 3C indicate that all the mutant proteins and the wild-type protein are expressed at a similar level in the transfected cell lysates. The data have thus provided a control showing that any change in the localization of a mutant protein in *T. brucei* shown below could not be attributed to an altered protein level.

Fluorescence microscopy demonstrated that ΔPB1-EYFP was associated with the FAZ among all the cells expressing this protein, ΔPB2-EYFP and ΔLinker-EYFP were found associated with the FAZ among 83% and 56% of the transfected cells, respectively, whereas no ΔPB1+2-EYFP was found localized to the FAZ among any of the transfected cells under examination (Figure 4A & Table 1). The association of mutant proteins with FAZ was able to sustain the harsh detergent and high salt treatment as the wild-type protein (Figure 4B, arrows), indicting a similar mechanism of binding to the FAZ among these proteins.

The two polo boxes are thus apparently playing the essential role of localizing TbPlk to the FAZ, while presence of the linker region between the two boxes could be also beneficial for an optimal binding of TbPlk to the FAZ. Presence of either PB1 or PB2 alone still enables the protein to bind to FAZ, apparently in a less efficient manner, because some of the proteins are left in the cytoplasm (Figure 4A). The partial failure of ΔLinker-EYFP and the total failure of ΔPB1+2-EYFP in localizing to the FAZ also leave substantial amount of the proteins in the cytoplasm as would have been anticipated (Figure 4A & Table 1).

An additional interesting observation made from the fluorescence microscopic experiments was that, while no wild-type TbPlk-EYFP, ΔPB2-EYFP or ΔLinker-EYFP protein was detected in the nucleus of transfected cells, about 80% of the cells expressing ΔPB1-EYFP or ΔPB1+2-EYFP had the protein localized also in the nucleus. The two mutants have relatively intact kinase activities. The common feature between the two is the deletion of PB1, suggesting that other than the kinase activity, the presence of PB1 provides also another block preventing TbPlk from entering the nucleus (see Discussion).

## Discussion

The unique subcellular localization of TbPlk to the FAZ and its limited function of regulating only the cytokinesis in *T. brucei* have provided an interesting opportunity for further investigation of the evolving mechanisms of cell cycle regulation. Since our previous indication that TbPlk was capable of complementing the multiple functions of Cdc5 in yeast [27], our current finding that Cdc5 localizes to the FAZ in *T. brucei* provides a clear indication that the discrepant functions and localizations of TbPlk and Cdc5 are not attributed to the differences between the two proteins *per se*. It could be attributed to the different cellular environments in trypanosome and yeast. The same enzyme protein may encounter different substrates or binding partners. Or, they could become substrates to different kinases in two different cells resulting in distintive subcellular localizations and functions.

Our experimental results showed also that the kinase activity of TbPlk does not play an essential role in localizing itself to the FAZ, nor is the entire kinase domain in TbPlk required for that purpose. The discrepancies between the primary structures of TbPlk and Cdc5 (see Figure S1) are apparently inconsequential in determining their locations in either *T. brucei* or *S. cerevisiae*. For instance, W517,V518 and L530 in the PB1 of Cdc5 have been identified to be the critical residues for localizing Cdc5 to the spindle poles and cytokinetic neck filaments in yeast [11]. These residues are missing from the PB1 of TbPlk. But the fact that the latter can still assume the multiple functions of Cdc5 in yeast suggests that TbPlk is likely localized also at the spindle poles and cytokinetic neck filaments of yeast. It is thus probably the secondary or tertiary structure of the polo boxes in the two proteins that plays pivotal roles in determining their subcellular localizations.

The additional experimental data indicated that the PBD, which consists of PB1 and PB2 separated by the linker region in TbPlk, provides the essential as well as perhaps the sufficient structural motif for binding to the FAZ in *T. brucei*. Since Cdc5 expressed in *T. brucei* also localizes to the FAZ, it is tempting to assume that the PBD in Cdc5 is also instrumental in directing the yeast kinase to FAZ, even though the two PBDs share only ∼40% sequence similarity (Figure S1). This PBD-dependent spatial regulation of Plks has been recognized in other living organisms [7]. A dominant negative human Plk1 PBD is sufficient to interfere with the subcellular localization and mitotic functions of Plk1 [17], [18]. Thus, PBD-dependent protein-protein interactions are probably playing an important role in Plk1 localization, presumably through binding to specific phosphorylated sequenses or unphosphorylated sequences in the target proteins [5]. By a combined biochemical and proteomic approach on Plk1, numerous phospho-dependent and phospho-independent PBD-binding proteins have been identified [19], though their individual involvement, if any, in Plk1 spatial regulation remains unknown. Many homologues of these binding proteins were identified also in the trypanosome genomic database (data unpublished). However, if one assumes that the protein binding to the PBD of TbPlk must be associated with FAZ for localizing the kinase to the latter, no such protein could be identified using the data on Plk1 at the present time. FAZ has a filamentous structure consisting of a specialized microtubule quartet and a unique area of flagellum:plasma membrane attachment that is resistant to detergent and high salt treatment [33]. The only protein identified in FAZ thus far is FAZ1. A knockdown of FAZ1 by RNAi resulted in compromised FAZ assembly and defects in flagellum attachment and cytokinesis [33]. But FAZ1 is not a homologue of any of the proteins known to bind to the PBD in Plk1 [7].

A bilobed structure was recently identified in *T. brucei* [34]. It is associated with the Golgi and the basal body, the origin of FAZ and flagellum. Duplication of the bilobe leads to duplication of Golgi, growth of the new FAZ and the new flagellum and initiation of cytokinesis [30]. TbPlk was found localized in the bilobe upon its early emergence. It then migrates along the growing tip of the new FAZ [30], and stops at the mid-point of FAZ while the cells reach anaphase. This is followed by a diffusion of TbPlk from the FAZ into the cytoplasm before cytokinetic initiation [31]. There have been two centrin homologues, TbCentrin2 [34] and TbCentrin1 [35], a membrane occupation and recognition nexus-containing protein (TbMORN) [36] and a homologue of leucine-rich repeats containing protain (TbLRRP1) recently identified in the bilobe structure of *T. brucei* [37], but they are associated with the bilobe throughout the cell cycle. TbCentrin2 is a substrate of TbPlk [30], whereas TbLRRP1 is a homologue of a spindle associated protein Nud1 in yeast that is phosphorylated by Cdc5 [38]. These two proteins may be phosphrylated by TbPlk inside the bilobe. However, since the kinase activity of TbPlk is not required for its FAZ localization, the phosphorylated TbCentrin2 and TbLRRP1may not be required for moving TbPlk to the FAZ. But the possibility remains that unphosphorylated TbCentrin and TbLRRP1 may recruit TbPlk to the bilobe by binding to its PBD. Further studies will be necessary for identifying the potential binding partners of TbPlk to localize it to the FAZ.

There is a putative bipartite nuclear localization signal (NLS) next to the C-terminal end of the kinase domain in TbPlk (***RRR***QHSDDPRGHAQGPLPL***RRQK***, residue 315–337, see Figure 2 and Figure S1). It is apparently non-functional in *T. brucei*, since TbPlk localizes exclusively to the FAZ. In mammalian cells, Plk1 has a functional bipartite NLS between residues 134–146 (***RRR***SLLELH***KRRK***) in the KD region that is necessary and sufficient for directing nuclear localization of Plk1 [32]. It is thus somewhat puzzling why the putative NLS in TbPlk does not function.

Data from some of the TbPlk mutants may have provided some of the answers to this question. ΔKD, N169A, ΔPB1 and ΔPB1+2 all demonstrated substantial presence in the nucleus, whereas ΔPB2 and ΔLinker were still being kept out of the nucleus like the wild type protein (Table 1). These interesting results may lead to two tentative conclusions; (1) The kinase activity of TbPlk could be instrumental in keeping the protein from the nucleus, presumably by phosphorylating certain key protein(s); (2) Even when the kinase activity remains unchanged (see Figure 3), deletion of PB1 allows nuclear import of the protein. It is possible that the loss of PB1 leads to failure of binding the kinase protein to the substrate(s), whose phosphorylation may prevent the nuclear import of TbPlk. Alternatively, since PB1 is localized downstream from the C-terminal end of the presumed NLS, its removal may activate the NLS from the cryptic state to an active state, which likely relies on a three-dimensional structural change of TbPlk. The nuclear import of a kinase inactive TbPlk mutant may not be of much biological interest. But it would be extremely interesting to see if the nuclear import of kinase-active ΔPB1 mutant of TbPlk will interfere with the mitotic regulation in *T. brucei*.

## Materials and Methods

### Cell culture

Procyclic-form *T. brucei* cells strain 29–13 were grown at 26°C in Cunningham's medium supplemented with 10% fetal bovine serum (HyClone). To maintain the T7 RNA polymerase and tetracycline repressor gene constructs within the cells, 15 µg/ml G418 and 50 µg/ml hygromycin B were added to the Cunningham's medium, respectively. Cells are maintained at mid-log phase through serial dilitions.

### Plasmids and cell lines

Plasmid expressing TbPlk tagged with EYFP was constructed from pLew100-TbPlk-3HA by replacing the 3HA with an EYFP tag [28] while the constrcution of pLew100-Cdc5-EYFP was generated by relacing the TbPlk gene in pLew100-TbPlk-EYFP with Cdc5 gene from pRS315-Cdc5 plasmid (a gift from Dr. Kevan Shokat, University of California, San Francisco) using the Afl II and Hind III restriction sites. The other TbPlk mutants were generated using the wild type TbPlk-EYFP as a template and created by using the ‘QuickChange’ site-directed mutagenesis kit from Stratagene (cat # 200521). Truncation muntant ΔPB1 was generated by deleting polo box 1 (residues from 564 to 642), ΔPB2 by deleting the residues from 690 to 756; ΔLinker by deleting residues 643 to 689 between the two polo boxes; ΔPB1+2 was obtained from deleting both polo boxes 1 and 2 but keeping the linker between them. All mutants were confirmed by sequencing after construction.

### Overexpression of Cdc5-EYFP, TbPlk-EYFP and its mutants

To over-express Cdc5-EYFP, TbPlk-EYFP and its mutants, the contructs for expressing the various fusoin proteins was each transfected into the cells. Briefly, 10^7^cells were suspended in 0.5 ml of cytomix buffer [39] after being washed with the same buffer, and mixed with 20 µg of a plasmid linearized with Not I. Electroporation was carried out in a 2-mm cuvette using the Gene Pulser (Bio-Rad) with parameters setting as follows: 1.6 kV voltage, 200 ohms resistance, and 25 microfarads capacitance. After electroporation, the cells were transferred to 10 ml of prewarmed Cunningham's medium immediately and incubated at 26°C for 24 h. Then Stable transfectants were selected under 2.5 µg/ml phleomycin. Expression of protein was induced by adding 1.0 µg/ml of tetracycline to the culture medium. Cell growth was monitored daily under a microscope with a hemacytometer.

### Western blotting

After being washed twice with phosphate-buffered saline (PBS), cells were boiled for 10 mins in sodium dodecyl sulfate-polyacrylamide gel electrophoresis (SDS-PAGE) sample buffer (Bio-Rad), and centrifuged at 1, 3000 rpm for 10 min. The protein sample was then separated by SDS-PAGE and transferred onto immunoblot polyvinylidene difluoride membrane (Bio-Rad). The membrane was blocked with TBST (20 mM Tris-HCl [pH 7.4], 150 mM NaCl, 0.1% Tween 20) containing 5% milk for 1 h and then incubated for 1 h with the antibody anti-green fluorescent protein (GFP) MAb JL-8 at a dilution ratio of 1∶5,000 (Clontech Laboratory Inc.) or anti-α-tubulin at a dilution ratio of 1∶10,000 (Sigma-Aldrich, MO). Anti-mouse immunoglobulin G (IgG) conjugated with horseradish peroxidase at a dilution ratio of 1∶10,000 (Sigma-Aldrich, MO) was used as the secondary antibody.

### Immunoprecipitation of Cdc5-EYFP, TbPlk-EYFP and its mutants and the kinase assay

Fusion proteins were each immunoprecipitaed with GFP-trap beads (Chromotek) from cell lysate. *T. brucei* lysate was prepared by incubating the cells with lysis buffer (25 mM Tris-Cl, pH7.6, 100 mM NaCl, 1 mM DTT, 1% Nonidet P-40 and protease inhibitor cocktail) on ice for 30 min and cleared by centrifugation [40]. GFP-trap beads was equilibrated with lysis buffer and blocked with 10 mg/ml BSA (New England Biolab) before being incubated with the cell lysate. After three washes with buffer (10 mM Tris/Cl, pH 7.5, 150 mM NaCl and protease inhibitor cocktail), the protein pulled down with the beads (Invitrogen) was incubated with 10 µg dephosphorylated casein (Sigma-Aldrich) in the presence of 15 mM HEPES (pH 7.5), 2 mM dithiothreitol, 20 mM MgCl_2_, 25 µM ATP, and 5 μCi [γ-^32^P] ATP for 1 hour at 37°C. The reaction was stopped by adding 5×SDS sample buffer to the assay mixture and boiled for 5 min. Proteins in the reaction mixture were separated by SDS-PAGE and exposed to a PhosphorImager for radiolabeled casein detection [31]. To get a equal loading of each kinase sample in the kinase assay, each immunoprecipitated sample was quantified in Western blot stained with anti-GFP (Clontech).

### Fluorescence microscopy

Cells expressing proteins tagged with EYFP were collected by centrifugation at 3000 rpm for 3 min, washed once in PBS, and fixed with 3.7% formaldehyde. The fixed cells were washed again with PBS, suspended in PBS, and allowed to adhere to poly-L-lysine-treated coverslips. The slides were mounted in VectaShield mounting medium containing 4′,6′-diamino-2-phenylindole (DAPI) and examined with a fluorescence microscope.

### Immunofluorescence staining

After being washed twice with PBS, cells were loaded on poly-L-lysine coated coverslips for 20 min and then washed three times with PEME buffer [100 mM piperazine-N,N -bis(2-ethanesulfonic acid) sesquisodium (PIPES 1.5 Na), 2 mM EGTA, 0.1 mM EDTA, and 1 mM MgSO_4_]. After the third wash, PEME buffer containing 1% NP-40 was added at room temperature for 5 min and the cells were rewashed with PEME buffer for a cytoskeleton preparation. The cytoskeleton was then fixed using 3.7% formaldehyde and blocked in 5% bovine serum albumin in PBS. After blocking, the sample was incubated with primary antibody L3B2 to FAZ (mouse MAb from Keith Gull, Oxford University; used at a dilution of 1∶50) [28] for one hour, and washed three times with PBS. The anti-mouse IgG conjugated with Atto 647N (Sigma-Aldrich) was used as the secondary antibody and incubated with the cells for another hour. Cells were then washed three times with PBS. The slides were mounted in Vectashield mounting medium containing 4′,6′-diamino-2-phenylindole (DAPI) (Vector Laboratories Inc.) and examined with a fluorescence microscope (ECLIPSE Ti; Nikon).

## Supporting Information

Figure S1 **An alignment of the protein sequences between TbPlk and Cdc5.**
(TIF)

## References

[1] Archambault V, Glover DM (2009). Polo-like kinases: conservation and divergence in their functions and regulation.. Nat Rev Mol Cell Biol.

[2] Dai W (2005). Polo-like kinases, an introduction.. Oncogene.

[3] Barr FA, Sillje HH, Nigg EA (2004). Polo-like kinases and the orchestration of cell division.. Nat Rev Mol Cell Biol.

[4] Lowery DM, Lim D, Yaffe MB (2005). Structure and function of Polo-like kinases.. Oncogene.

[5] Elia AE, Cantley LC, Yaffe MB (2003). Proteomic screen finds pSer/pThr-binding domain localizing Plk1 to mitotic substrates.. Science.

[6] Li B, Ouyang B, Pan H, Reissmann PT, Slamon DJ (1996). Prk, a cytokine-inducible human protein serine/threonine kinase whose expression appears to be down-regulated in lung carcinomas.. J Biol Chem.

[7] Park JE, Soung NK, Johmura Y, Kang YH, Liao C (2010). Polo-box domain: a versatile mediator of polo-like kinase function.. Cell Mol Life Sci.

[8] Elia AE, Rellos P, Haire LF, Chao JW, Ivins FJ (2003). The molecular basis for phosphodependent substrate targeting and regulation of Plks by the Polo-box domain.. Cell.

[9] Kelm O, Wind M, Lehmann WD, Nigg EA (2002). Cell cycle-regulated phosphorylation of the Xenopus polo-like kinase Plx1.. J Biol Chem.

[10] Jang YJ, Ma S, Terada Y, Erikson RL (2002). Phosphorylation of threonine 210 and the role of serine 137 in the regulation of mammalian polo-like kinase.. J Biol Chem.

[11] Song S, Grenfell TZ, Garfield S, Erikson RL, Lee KS (2000). Essential function of the polo box of Cdc5 in subcellular localization and induction of cytokinetic structures.. Mol Cell Biol.

[12] Lee KS, Grenfell TZ, Yarm FR, Erikson RL (1998). Mutation of the polo-box disrupts localization and mitotic functions of the mammalian polo kinase Plk.. Proc Natl Acad Sci U S A.

[13] Jang YJ, Lin CY, Ma S, Erikson RL (2002). Functional studies on the role of the C-terminal domain of mammalian polo-like kinase.. Proc Natl Acad Sci U S A.

[14] Jiang N, Wang X, Jhanwar-Uniyal M, Darzynkiewicz Z, Dai W (2006). Polo box domain of Plk3 functions as a centrosome localization signal, overexpression of which causes mitotic arrest, cytokinesis defects, and apoptosis.. J Biol Chem.

[15] Archambault V, D'Avino PP, Deery MJ, Lilley KS, Glover DM (2008). Sequestration of Polo kinase to microtubules by phosphopriming-independent binding to Map205 is relieved by phosphorylation at a CDK site in mitosis.. Genes Dev.

[16] Ma S, Liu MA, Yuan YL, Erikson RL (2003). The serum-inducible protein kinase Snk is a G1 phase polo-like kinase that is inhibited by the calcium- and integrin-binding protein CIB.. Mol Cancer Res.

[17] Hanisch A, Wehner A, Nigg EA, Sillje HH (2006). Different Plk1 functions show distinct dependencies on Polo-Box domain-mediated targeting.. Mol Biol Cell.

[18] Seong YS, Kamijo K, Lee JS, Fernandez E, Kuriyama R (2002). A spindle checkpoint arrest and a cytokinesis failure by the dominant-negative polo-box domain of Plk1 in U-2 OS cells.. J Biol Chem.

[19] Lowery DM, Clauser KR, Hjerrild M, Lim D, Alexander J (2007). Proteomic screen defines the Polo-box domain interactome and identifies Rock2 as a Plk1 substrate.. EMBO J.

[20] Cheng KY, Lowe ED, Sinclair J, Nigg EA, Johnson LN (2003). The crystal structure of the human polo-like kinase-1 polo box domain and its phospho-peptide complex.. EMBO J.

[21] Leung GC, Hudson JW, Kozarova A, Davidson A, Dennis JW (2002). The Sak polo-box comprises a structural domain sufficient for mitotic subcellular localization.. Nat Struct Biol.

[22] Liao CZ, Park JE, Bang JK, Nicklaus MC, Lee KS (2010). Probing Binding Modes of Small Molecule Inhibitors to the Polo-Box Domain of Human Polo-like Kinase 1.. ACS Med Chem Lett.

[23] Sherwin T, Gull K (1989). The cell division cycle of Trypanosoma brucei brucei: timing of event markers and cytoskeletal modulations.. Philos Trans R Soc Lond B Biol Sci.

[24] Moreira-Leite FF, Sherwin T, Kohl L, Gull K (2001). A trypanosome structure involved in transmitting cytoplasmic information during cell division.. Science.

[25] Li Z, Lee JH, Chu F, Burlingame AL, Gunzl A (2008). Identification of a novel chromosomal passenger complex and its unique localization during cytokinesis in Trypanosoma brucei.. PLoS One.

[26] Graham TM, Tait A, Hide G (1998). Characterisation of a polo-like protein kinase gene homologue from an evolutionary divergent eukaryote, Trypanosoma brucei.. Gene.

[27] Kumar P, Wang CC (2006). Dissociation of cytokinesis initiation from mitotic control in a eukaryote.. Eukaryot Cell.

[28] Umeyama T, Wang CC (2008). Polo-like kinase is expressed in S/G2/M phase and associated with the flagellum attachment zone in both procyclic and bloodstream forms of Trypanosoma brucei.. Eukaryot Cell.

[29] Hammarton TC, Kramer S, Tetley L, Boshart M, Mottram JC (2007). Trypanosoma brucei Polo-like kinase is essential for basal body duplication, kDNA segregation and cytokinesis.. Mol Microbiol.

[30] de Graffenried CL, Ho HH, Warren G (2008). Polo-like kinase is required for Golgi and bilobe biogenesis in Trypanosoma brucei.. J Cell Biol.

[31] Li Z, Umeyama T, Wang CC (2010). Polo-like kinase guides cytokinesis in Trypanosoma brucei through an indirect means.. Eukaryot Cell.

[32] Taniguchi E, Toyoshima-Morimoto F, Nishida E (2002). Nuclear translocation of plk1 mediated by its bipartite nuclear localization signal.. J Biol Chem.

[33] Vaughan S, Kohl L, Ngai I, Wheeler RJ, Gull K (2008). A repetitive protein essential for the flagellum attachment zone filament structure and function in Trypanosoma brucei.. Protist.

[34] He CY, Pypaert M, Warren G (2005). Golgi duplication in Trypanosoma brucei requires Centrin2.. Science.

[35] Selvapandiyan A, Kumar P, Morris JC, Salisbury JL, Wang CC (2007). Centrin1 is required for organelle segregation and cytokinesis in Trypanosoma brucei.. Mol Biol Cell.

[36] Morriswood B, He CY, Sealey-Cardona M, Yelinek J, Pypaert M (2009). The bilobe structure of Trypanosoma brucei contains a MORN-repeat protein.. Mol Biochem Parasitol.

[37] Zhou Q, Gheiratmand L, Chen Y, Lim TK, Zhang J (2010). A comparative proteomic analysis reveals a new bi-lobe protein required for bi-lobe duplication and cell division in Trypanosoma brucei.. PLoS One.

[38] Park CJ, Park JE, Karpova TS, Soung NK, Yu LR (2008). Requirement for the budding yeast polo kinase Cdc5 in proper microtubule growth and dynamics.. Eukaryot Cell.

[39] van den Hoff MJ, Moorman AF, Lamers WH (1992). Electroporation in ‘intracellular’ buffer increases cell survival.. Nucleic Acids Res.

[40] Li Z, Umeyama T, Wang CC (2008). The chromosomal passenger complex and a mitotic kinesin interact with the Tousled-like kinase in trypanosomes to regulate mitosis and cytokinesis.. PLoS One.

