# Supplementary figures and images for: The Structural Basis of Localizing Polo-Like Kinase to the Flagellum Attachment Zone in Trypanosoma brucei
Source: PLoS One. 2011 Nov 11;6(11):e27303. doi: 10.1371/journal.pone.0027303 (PMC3214037; doi:10.1371/journal.pone.0027303)

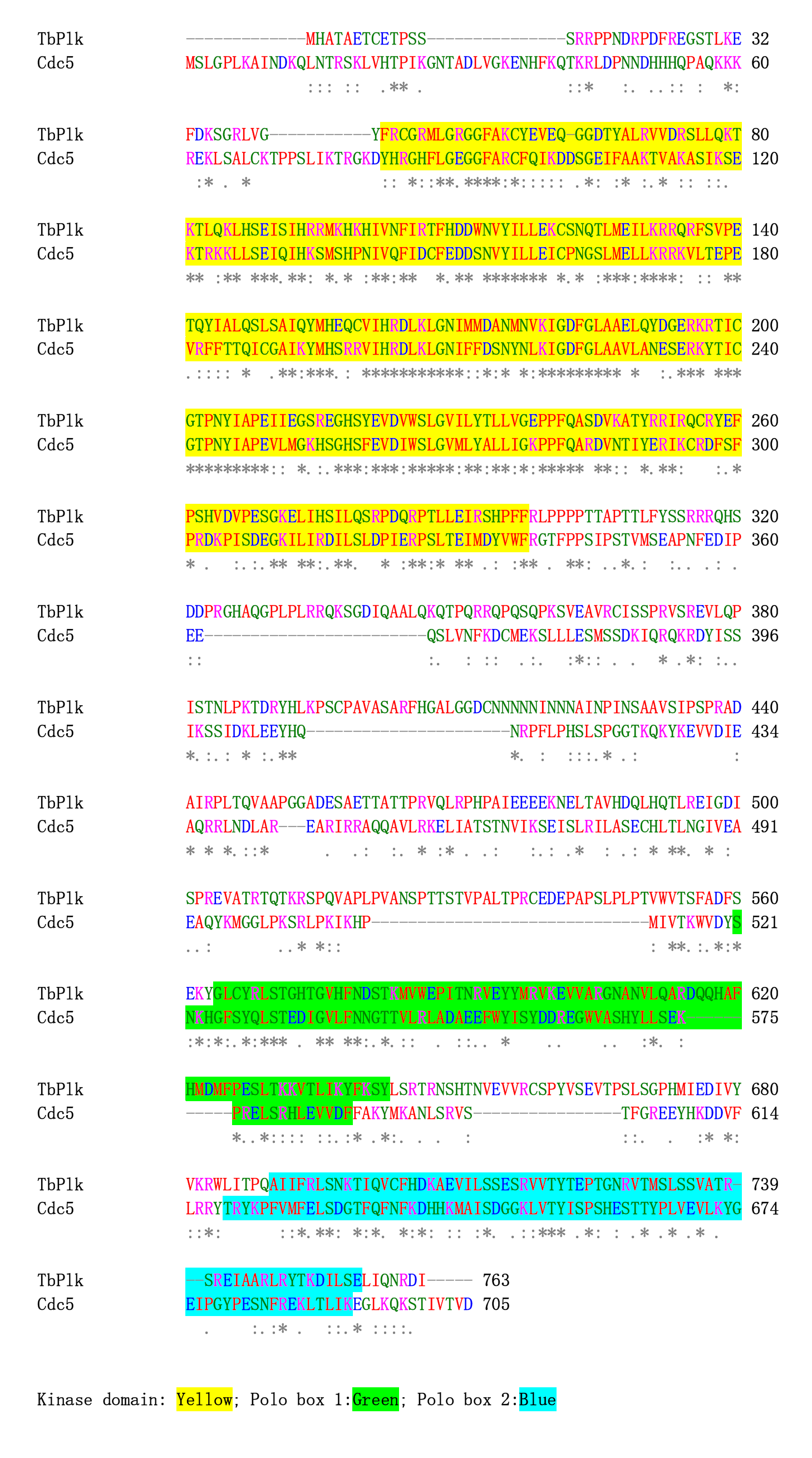

Supplement: Figure S1 — An alignment of the protein sequences between TbPlk and Cdc5. (TIF) [file pone.0027303.s001.tif]
